# Systematic review: probiotics in the management of lower gastrointestinal symptoms in clinical practice – an evidence-based international guide

**DOI:** 10.1111/apt.12460

**Published:** 2013-08-27

**Authors:** A P S Hungin, C Mulligan, B Pot, P Whorwell, L Agréus, P Fracasso, C Lionis, J Mendive, J-M Philippart de Foy, G Rubin, C Winchester, N Wit

**Affiliations:** * School of Medicine, Pharmacy and Health, Durham UniversityStockton-on-Tees, UK; † Research Evaluation Unit, Oxford PharmaGenesis™ LtdOxford, UK; ‡ Institut Pasteur de Lille, Centre for Infection and Immunity of LilleLille, France; § Université Lille Nord de FranceLille, France; ¶ CNRS UMR 8204Lille, France; ** INSERM U1019Lille, France; †† Centre for Gastrointestinal Sciences, University of Manchester, Wythenshawe HospitalManchester, UK; ‡‡ Centre for Family Medicine, Karolinska InstituteStockholm, Sweden; §§ Gastroenterology Unit, Don Bosco Outpatient ClinicRome, Italy; ¶¶ Clinic of Social and Family Medicine, School of Medicine, University of CreteHeraklion, Greece; *** La Mina Primary Care CentreBarcelona, Spain; ††† Nutrition Committee of the Scientific Society of General Practice (SSMG, Belgium)Brussels, Belgium; ‡‡‡ Julius Centre for Health Sciences and Primary Care, UMC UtrechtUtrecht, The Netherlands

## Abstract

**Background**Evidence suggests that the gut microbiota play an important role in gastrointestinal problems.

**Aim**To give clinicians a practical reference guide on the role of specified probiotics in managing particular lower gastrointestinal symptoms/problems by means of a systematic review-based consensus.

**Methods**Systematic literature searching identified randomised, placebo-controlled trials in adults; evidence for each symptom/problem was graded and statements developed (consensus process; 10-member panel). As results cannot be generalised between different probiotics, individual probiotics were identified for each statement.

**Results**Thirty seven studies were included; mostly on irritable bowel syndrome [IBS; 19 studies; treatment responder rates: 18–80% (specific probiotics), 5–50% (placebo)] or antibiotic-associated diarrhoea (AAD; 10 studies). Statements with 100% agreement and ‘high’ evidence levels indicated that: (i) specific probiotics help reduce overall symptom burden and abdominal pain in some IBS patients; (ii) in patients receiving antibiotics/*Helicobacter pylori* eradication therapy, specified probiotics are helpful as adjuvants to prevent/reduce the duration/intensity of AAD; (iii) probiotics have favourable safety in patients in primary care. Items with 70–100% agreement and ‘moderate’ evidence were: (i) specific probiotics help relieve overall symptom burden in some patients with diarrhoea-predominant IBS, and reduce bloating/distension and improve bowel movement frequency/consistency in some IBS patients and (ii) with some probiotics, improved symptoms have led to improvement in quality of life.

**Conclusions**Specified probiotics can provide benefit in IBS and antibiotic-associated diarrhoea; relatively few studies in other indications suggested benefits warranting further research. This study provides practical guidance on which probiotic to select for a specific problem.

## Introduction

Gastrointestinal (GI) problems are a major reason for consultation.^1^ Symptom management of GI problems often begins in primary care with adjustment of lifestyle factors that may cause or worsen symptoms, such as diet.^2^ Pharmacological treatments for patients with functional GI disorders (FGID) have limited efficacy and may cause side effects.^3^–^4^ Given that changes in the gut microbiota have been implicated in the pathogenesis of GI disorders [such as irritable bowel syndrome (IBS)],^5^–^8^ there is growing interest in therapies that might influence these changes, such as probiotics.

Probiotics are defined as ‘live microorganisms which when administered in adequate amounts confer a health benefit on the host'.^9^ These are distinct from prebiotics (dietary substances such as indigestible oligosaccharides that provide a health benefit by selectively promoting the growth of beneficial bacteria in the gut) and synbiotics (products containing a synergistic combination of prebiotics and probiotics). The remainder of this article will focus on probiotics. Despite their long history, wide availability and substantial publication record, the clinical role of probiotics has, in general, been inadequately characterised and remains ill-defined. Attempts to summarise probiotic research are complicated by the wide variety of probiotic strains that are available, as results obtained with one strain are not generalisable to others.^10^ The range of different formulations (capsules, sachets, yoghurts and fermented milks or fruit drinks), the dose and the presence of supporting substrates add further sources of variation.^11^,^12^ Effects, moreover, may be different according to age and health status of the target group.^14^–^15^

Many gastroenterologists recommend probiotics,^16^–^17^ and primary care physicians are increasingly confronted with questions about the suitability (or otherwise) of probiotics, but their familiarity with probiotics is limited.^18^–^19^ All clinicians are faced with an increasingly broad range of products, and deciding whether or not to recommend one of these to a particular patient is a major challenge. At the same time, the public is exposed to widespread claims for probiotics with a variety of products in shops, without clear guidance as to which might be useful. Clear, evidence-based guidance is therefore needed regarding the effectiveness of different probiotics and their clinical use.

Clinical guidelines usually focus on specific disease entities, but primary care physicians and gastroenterologists working in the field of FGID generally have to deal with overlapping symptom complexes.^20^ Consequently, the aim of this study was to provide practical advice to clinicians regarding the use of probiotics in the treatment of lower GI symptoms in adults in clinical practice. This advice was based on an extensive review of the literature followed by a validated approach to developing consensus that crosses international boundaries. The findings were translated into a reference tool identifying available probiotics with evidence for/against a beneficial effect for different GI symptoms/problems, to help clinicians make appropriate, evidence-based treatment decisions.

## Methods

### Systematic literature searches

**Figure 1:**
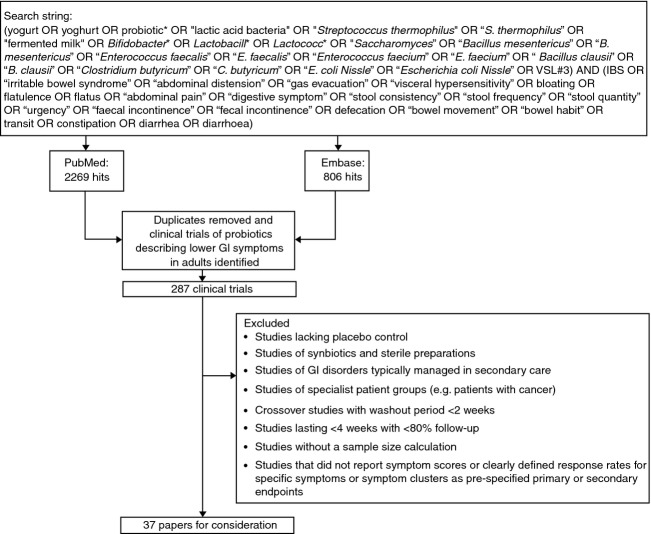
Flow diagram of literature searches. The initial PubMed and Embase searches were performed on 31 January 2012, and were limited to English language publications. GI, gastrointestinal.

Systematic literature searches were performed (based on AGREE II criteria^21^) to answer the following question: in high-quality clinical studies performed in adults, what effects do probiotics have on lower GI symptoms/problems that are typically managed in primary care? PubMed and Embase (which together provide extensive coverage of the biomedical literature) were searched to identify all studies that assessed the effect of probiotics on lower GI symptoms, using the search string shown in Figure 1. The search results were combined and duplicates were removed. An initial screen of article titles and abstracts was then performed to identify clinical trials of probiotics that studied lower GI symptoms in adults (≥18 years old). Only studies of adults were included because the intestinal microbiota differ between children and adults.^14^ In addition, trials that evaluated only synbiotics were excluded. Studies of patients with IBS or other FGID; diarrhoea as a side effect of antibiotic treatment; lactose intolerance; or no specific GI diagnosis were included. Studies of well-defined disorders such as inflammatory bowel disease and diverticular disease were excluded. Studies of specialist populations (e.g. patients with any type of cancer) were also excluded.

The output of the systematic literature searches was discussed by the Consensus Group in a face-to-face workshop. To ensure a high-quality evidence base, they agreed to exclude the following: (i) studies without a placebo control group; (ii) crossover studies with a washout period of less than 2 weeks; (iii) studies in which fewer than 80% of participants were followed up unless the study duration exceeded 4 weeks; (iv) studies that did not perform a sample size calculation; (v) studies that did not report symptom scores or clearly defined response rates for specific symptoms or symptom clusters as prespecified primary or secondary end points. Prespecified primary/secondary end points were to be listed as such in the section or in the study objectives at the end of the section of the article under consideration.

Data for the following lower GI symptoms/problems were extracted from the included articles: IBS; abdominal pain; bloating/distension; flatus; constipation; bowel habit (e.g. frequency and/or consistency of bowel movements); diarrhoea (as part of IBS or associated with use of antibiotics including *Helicobacter pylori* eradication therapy). Health-related quality of life data were also extracted. As it was evident from previous publications that different probiotic strains will have different effects,^10^ the identities of the probiotic strains used in each study were recorded. Results of adverse event monitoring were also recorded if available.

### Consensus development

A modified Delphi process was used to develop consensus statements. The Delphi process is an increasingly widely used technique for reaching expert consensus.^22^,^23^ It uses a process of anonymous and iterative feedback and voting to achieve consensus among a panel of independent experts by means of stepwise refinement of responses.

The Consensus Group consisted of primary care physicians with an interest in gastroenterology drawn from the European Society for Primary Care Gastroenterology (ESPCG), with the addition of one primary care physician from Belgium, two members from secondary care and a microbiologist. The Group was led by a nonvoting Chair (APSH, ESPCG Research Officer) who, in common with other members of the Consensus Group, has experience of systematic reviews and guideline development. Statements were developed (based on evidence and clinical experience) by the Chair in collaboration with a Steering Committee (BP, NdW and PW).

### Development and grading of statements

Statements were prepared for each of the categories outlined above. The level of supporting evidence and strength of each statement were rated by the Chair and Steering Committee using the Grades of Recommendation Assessment, Development and Evaluation (GRADE) system^25^ as follows: high – further research is unlikely to change our confidence in the estimate of effect; moderate – further research is likely to have an important impact on our confidence in the estimate of effect and may change the estimate; low – further research is very likely to have an important impact on our confidence in the estimate of effect and is likely to change the estimate; and very low – any estimate of effect is very uncertain.

### Refinement of statements

The statements were worded to reflect the grade of available evidence. For example, the phrase ‘probiotics **may** help to…’ was used to distinguish statements with a low grade of evidence from those with a moderate or high grade of evidence (‘probiotics help to…’). The proportion of patients with IBS who responded to treatment did not exceed 80% in the included studies (despite ‘responders’ being defined very broadly as patients showing any improvement from baseline in some studies). Therefore, statements relating to potential benefits in IBS used the phrase ‘in **some** patients with IBS’ rather than ‘in patients with IBS’. Two rounds of anonymous voting on the statements were performed. Votes were cast using an online platform (INSINC Consulting, Guelph, ON, Canada and ECD Solutions, Atlanta, GA, USA), with each round being analysed by the nonvoting Chair (APSH). For each statement, voters indicated their level of agreement on a scale from 1 to 6 (1: strongly disagree; 2: disagree with major reservation; 3: disagree with minor reservation; 4: agree with major reservation; 5: agree with minor reservation; and 6: strongly agree). Consensus was defined *a priori* as agreement by at least 67% of respondents.

## Results

In total, 37 publications were identified and used to develop statements. Table 1 provides a summary of the symptoms and indications examined in the 37 studies, all of which had a placebo control group or placebo-controlled period. Collectively, the 37 studies investigated a total of 32 different probiotics at doses of 1 × 10^6^–4.5 × 10^11^ colony forming units (CFU) administered once, twice or three times daily. They predominantly contained bacteria (mostly lactobacilli and/or bifidobacteria); a few contained *Saccharomyces*. Of note, the term ‘probiotics’ has been used throughout this section to refer to products that contain probiotics, regardless of whether the product contains a single strain or multiple strains.

**Table 1:** Indications and symptoms examined in included studies

Number of studies	Indication						
Symptom	IBS	Functional GI disorders	Antibiotic treatment	*Helicobacter pylori* eradication	Lactose intolerance	Healthy/minor GI symptoms	Total
IBS (global symptoms)	16	0	0	0	0	0	16
Abdominal pain	18	2	0	0	1	2	23
Bloating/distension	15	1	0	0	1	2	19
Flatus	10	2	0	0	1	2	15
Diarrhoea (treatment)	3	2	0	0	1	1	7
Diarrhoea (prevention)	0	0	6	4	0	0	10
Constipation	2	2	0	0	0	0	4
Bowel habit	17	1	0	0	0	3	21
Health-related quality of life	12	1	0	0	0	2	15
Total	19	2	6	4	1	5	37

GI, gastrointestinal; IBS, irritable bowel syndrome.

Treatment adherence was addressed in 29 of the included studies. In 27 of the included studies, adherence to treatment was assessed by counting empty containers/unused test substance returned at the end of the study and/or by participant self-reporting (in treatment diaries or during investigator visits). Faecal recovery of probiotic strains was used as a measure of adherence in three of the studies. Where adherence data were reported (21 studies), the level of adherence was generally high. In the active treatment groups, the proportion of participants who were adherent to treatment was >75–100%. In the faecal recovery analyses, 79–92% of participants in the active treatment groups tested positive for the specific probiotic strain(s).

The majority of the studies focused on IBS (based on Rome I, II or III criteria or physician diagnosis; 19 studies) or antibiotic-associated diarrhoea (AAD; 10 studies, of which four examined *H. pylori* eradication therapy). The studies in IBS tended to include all IBS subtypes, with only two studies focusing on constipation-predominant IBS (IBS-C), and three studies focusing on diarrhoea-predominant IBS (IBS-D). The IBS studies employed different definitions of treatment response and reported a correspondingly wide range of ‘responder’ rates (18–80% and 5–50% in groups receiving specific probiotics and placebo respectively). Table 2 provides an overview of the 37 studies, including the indication studied, the probiotic treatment regimens used, study design and the number of patients analysed.

**Table 2:** Overview of probiotic treatment regimens and results in included studies

Diagnosis	Patients analysed (*n*)	Study design	Probiotic strain(s)* (brand name)Formulation and regimen	Primary end point	Results for primary end point	*P* value	Reference
**Marketed products**							
IBS (Rome II)	100	DBRCT	*Bifidobacterium longum* subsp. *longum* LA 101, *Lactobacillus acidophilus* LA 102, *L. delbrueckii* subsp. *lactis* LA 103, *Streptococcus salivarius* subsp. *thermophilus* LA 104 (Lactibiane)Sachets, 1 × 10^10^ CFU o.i.d. for 4 weeks	Satisfactory relief of overall IBS symptoms, and abdominal pain/discomfort score	Proportion with satisfactory relief: specific probiotic, 42.6%; placebo, 42.3%.Reduction in abdominal pain score from first to fourth week of treatment: specific probiotic, −41.9%; placebo, −24.2%	> 0.050.048	Drouault-Holowacz *et al*.^26^
IBS, including abdominal pain (diagnosed by a primary care physician)	298	DBRCT	*Escherichia coli* DSM17252 (Symbioflor-2)Oral liquid, 1.5–4.5 × 10^7^ CFU/mL for 8 weeks (0.75 mL t.i.d. for week 1; 1.5 mL t.i.d. for weeks 2–8)	Abdominal pain and overall IBS symptom scores (treatment response: absence of symptoms at ≥1 visit during treatment)	Abdominal pain response rate: specific probiotic, 18.9%; placebo, 6.7%Overall GI symptom response rate: specific probiotic, 18.2%; placebo, 4.7%	0.00160.0004	Enck *et al*.^27^
IBS (Rome II)	86	DBRCT	*L. rhamnosus* GG, *L. rhamnosus* Lc705, *Propionibacterium freudenreichii* subsp. *shermanii* JS, *B. animalis* subsp. *lactis* Bb12 (Gefilus MAX)Milk-based drink, 1.2 dL (1 × 10^7^ CFU/mL of each strain) o.i.d. for 5 months	Change in composite IBS symptom score (abdominal pain, distension, flatulence and rumbling)	Decrease from baseline: specific probiotic, 37%; placebo, 9%	0.0083	Kajander *et al*.^28^
IBS (Rome II) with abdominal bloating	48	DBRCT	*B. longum* subsp*. longum, B. infanti*s subsp*. infantis*,*B. breve*,*L. acidophilus*,*L. paracasei* subsp*. paracasei*,*L. delbrueckii* subsp. *bulgaricus*,*L. plantarum, Streptococcus salivarius* subsp. *thermophilus* (VSL#3)One sachet (450 billion lyophilised bacteria) b.i.d. for 4–8 weeks	Bloating severity score	Posttreatment score: specific probiotic, 31.3; placebo, 38.5	0.11	Kim *et al*.^29^
IBS (Rome criteria)	12	DBRCT, crossover	*L. plantarum* 299v (ProViva)Fermented oatmeal gruel, 6.25 × 10^9^ CFU o.i.d. (125 mL) for 4 weeks	Gas production	Gas production in 24 h: specific probiotic, 249 mL; placebo, 245 mL	>0.05	Sen *et al*.^30^
IBS (Rome II)	74	DBRCT	*L. paracasei* subsp. *paracasei* F19, *L. acidophilus* La5, *B. animalis* subsp. *lactis* Bb12 (Cultura)Fermented milk, 5 × 10^7^ CFU/mL, 200 mL b.i.d. for 8 weeks	Proportion of patients reporting adequate relief of their IBS symptoms at least 50% of the weeks during the treatment period (‘responders’)	Proportion of responders: specific probiotic, 38%; placebo, 27%	0.3	Simrén *et al*.^31^
IBS (Rome II)	52	DBRCT	*L. paracasei* subsp. *paracasei* F19, *L. acidophilus* La5, *B. animalis* subsp. *lactis* Bb 12 (Cultura)Fermented milk, 250 mL b.i.d. (7.5 × 10^10^ CFU/day) for 8 weeks	Proportion reporting adequate symptom relief, and total IBS-SSI score	No significant differences between specific probiotic and placebo	>0.05	Sondergaard *et al*.^32^
IBS (Rome II; females)	362	DBRCT	*B. longum* subsp. *infantis* 35624 (Bifantis/Align)Capsules, three dose groups: 1 × 10^6^, 1 × 10^8^ or 1 × 10^10^ CFU o.i.d. for 4 weeks	Abdominal pain/discomfort score	Change from baseline: −0.89 in the group receiving specific probiotic 1 × 10^8^ CFU o.i.d. compared to −0.58 in the placebo group	0.023	Whorwell *et al*.^33^
IBS (Rome II)	52	DBRCT	*L. acidophilus* (CUL60 and CUL21), *B. animalis* subsp. *lactis* CUL34, *B. bifidum* CUL20 (LAB4)Capsules, 2.5 × 10^10^ CFU o.i.d. for 8 weeks	IBS-SSI score	Difference (specific probiotic vs. placebo): 6 weeks: –47.82 8 weeks: –52.7310 weeks: no significant difference	0.03470.0217>0.05	Williams *et al*.^34^
IBS-C (Rome III)	34	DBRCT	*B. animalis* subsp. *lactis* DN-173 010 (Activia)Fermented milk, 125 g (1.25 × 10^10^ CFU) b.i.d. for 4 weeks	Abdominal distension (measured by abdominal inductance plethysmography)	Median percentage change in maximal distension (specific probiotic vs. placebo): −39%Mean abdominal distension (specific probiotic vs. placebo): −1.52 cm	0.020.096	Agrawal *et al*.^35^
IBS-C (Rome II)	267	DBRCT	*B. animalis* subsp. *lactis* DN-173 010 (Activia)Fermented milk, 125 g (1.25 × 10^10^ CFU) b.i.d. for 6 weeks	‘Discomfort’ dimension of the validated FDDQOL questionnaire (response: improvement of ≥ 10% vs. baseline)	Responder rate (week 3): specific probiotic, 65.2%; placebo, 47.7%Responder rate (week 6): specific probiotic, 63.0%; placebo, 56.8%)Change in score from baseline: no significant difference between groups	0.003>0.05>0.05	Guyonnet *et al*.^36^
IBS-D (Rome III)	50	DBRCT	*L. acidophilus* LH5*, L. plantarum* LP1, *L. rhamnosus* LR3, *B. breve* BR2, *B. animalis* subsp. *lactis* BL2, *B. longum* subsp*. longum* BG3, *Streptococcus salivarius* subsp. *thermophilus* ST3 (bacterial component of Duolac7)One capsule b.i.d. (1 × 10^10^ cells/day) for 8 weeks	Adequate relief of IBS symptoms for ≥50% of weeks during treatment and 2-week follow-up	Proportion with adequate symptom relief: specific probiotic, 48%; placebo, 12%	0.01	Ki Cha *et al*.^37^
IBS-D (Rome II)	25	DBRCT	*B. longum*,*B. longum* subsp. *infantis*,*B. breve*,*L. acidophilus*,*L. paracasei* subsp. *paracasei*,*L. delbrueckii* subsp. *bulgaricus*,*L. plantarum*,*Streptococcus salivarius* subsp. *thermophilus* (VSL#3)One sachet (225 billion lyophilised bacteria) b.i.d. for 8 weeks (total daily dose 450 billion bacteria)	Transit time and global satisfaction (treatment response: satisfactory relief of overall IBS symptoms on ≥4 of 8 weekly assessments)	GI transit: no difference between the two treatment groups.Proportion of responders: specific probiotic, 33%; placebo, 38%	0.41–0.991.00	Kim *et al*.^38^
IBS-D (Rome II)	29	SBRCT	*Streptococcus salivarius* subsp. *thermophilus* (1 × 10^8^ CFU/mL), *L. delbrueckii* subsp. *bulgaricus* (1 × 10^7^ CFU/mL), *L. acidophilus* (1 × 10^7^ CFU/mL), *B. longum* subsp*. longum* (1 × 10^7^ CFU/mL) (AB100 Jianneng)Fermented milk, 200 g b.i.d. for 4 weeks (each mL contained at least 1.3 × 10^8^ CFU total)	Improvement in proportion with abnormal intestinal permeability	N/A – primary end point data were not GI symptoms/HRQoL measures (IBS symptoms were assessed as secondary end points)	–	Zeng *et al*.^39^
AAD	89	DBRCT	*L. acidophilus* CL1285, *L. paracasei* subsp*. paracasei* LBC80R (Bio-K+ CL1285)Fermented milk, half container (49 g) o.i.d. for 2 days, then full container (98 g; 50 × 10^9^ CFU) o.i.d., starting within 48 h of initiating antibiotic treatment and continuing for duration of antibiotic treatment	AAD (≥3 liquid stools in a 24-h period)	Incidence of AAD: specific probiotic, 15.9%; placebo, 35.6%; OR, 0.343	0.05	Beausoleil *et al*.^40^
AAD	255	DBRCT	*L. acidophilus* CLl285, *L. paracasei* subsp*. paracasei* LBC80R (Bio-K+ CL1285)Capsules, two dose groups: One or two probiotic capsules (50 or 100 billion CFU)/day, initiated within 36 h of starting antibiotic treatment and continued for 5 days after completing antibiotic treatment (duration of antibiotic treatment was 3–14 days)	AAD (≥3 liquid stools in a 24-h period)	Incidence of AAD: specific probiotic (two capsules), 15.5%; specific probiotic (one capsule); 28.2%; placebo, 44.1%Duration of AAD: specific probiotic (two capsules), 2.8 days; specific probiotic (one capsule); 4.1 days; placebo, 6.4 days	≤0.02<0.001	Gao *et al*.^41^
AAD	437	DBRCT	*L. acidophilus* CL1285, *L. paracasei* subsp*. paracasei* LBC80R (Bio-K+ CL1285)Fermented milk, half container (49 g) o.i.d. for 2 days, then full container (98 g; 50 × 10^9^ CFU) o.i.d. for 29–40 days (started within 24 h after the first dose of antibiotic, and continued until 5 days after the last dose of antibiotic)	AAD (≥1 episodes of unformed or liquid stool in a 24-h period) severity and incidence	Mean number of days with AAD: specific probiotic, 0.67 days; placebo, 1.19 daysProportion of patients with AAD: specific probiotic, 21.8%; placebo, 29.4% (note study was underpowered).OR of AAD (multivariate logistic regression, specific probiotic vs. placebo): 0.627	0.0400.0670.037	Sampalis *et al*.^42^
AAD	113	DBRCT	*L. paracasei* subsp*. paracasei* DN-114 001 (*L. paracasei* subsp*. paracasei* immunitass) (Actimel)Yoghurt drink, 100 g (97 mL; 1 × 10^8^ CFU/mL) b.i.d. started within 48 h of starting antibiotic treatment and continued for 1 week after stopping antibiotic treatment (publication does not state duration of antibiotics). Follow-up was 4 weeks later	AAD (>2 liquid stools a day for ≥3 days in quantities in excess of normal for the patient)	Incidence of AAD: specific probiotic, 12%; placebo, 34%OR of diarrhoea (adjusted logistic regression, specific probiotic vs. placebo): 0.25	0.007	Hickson *et al*.^43^
AAD, *Clostridium difficile*-associated diarrhoea	138	DBRCT	*L. acidophilus* (CUL60 and CUL21), *B. animalis* subsp. *lactis* CUL34, *B. bifidum* CUL20 (LAB4; strains not given in publication; information obtained from company website)Capsules, 2 × 10^10^ CFU o.i.d. started within 36 h of antibiotic prescription (duration 20 days)	*C. difficile*-associated diarrhoea	N/A – *C. difficile*-associated diarrhoea was not covered in this consensus (AAD was assessed as a secondary end point)	–	Plummer *et al*.^44^
AAD	214	DBRCT	*L. rhamnosus* R0011, *L. acidophilus* R0052 (Lacidofil cap)One capsule (2 × 10^9^ CFU) b.i.d. starting within 48 h of initiating antibiotic treatment (duration 2 weeks)	AAD (loose or watery stools >3× per day for ≥2 days within 14 days of enrolment)	Incidence of AAD: specific probiotic, 3.9%; placebo, 7.2% (note study was underpowered)	0.44	Song *et al*.^45^
*H. pylori* therapy-associated side effects	124	DBRCT	*Saccharomyces boulardii* (Reflor)500 mg (two sachets) b.i.d. for 2 weeks during 2-week *H. pylori* eradication therapy. Patients were followed up for a further 2 weeks	*H. pylori* eradication therapy-associated side effects	Diarrhoea occurred in 14.5% of the specific probiotic group and 30.6% of the placebo group	<0.05	Cindoruk *et al*.^46^
*H. pylori* therapy-associated side effects	64	TBRCT	(1) *L. rhamnosus* GG (Giflorex)*Saccharomyces boulardii*† (Codex)(2) One sachet b.i.d. for 2 weeks (during and for 1 week after 1-week *H. pylori* eradication therapy). Each sachet contained 6 × 10^9^ CFU (1) or 5 × 10^9^ CFU (2)	*H. pylori* eradication therapy-associated side effects	Diarrhoea occurred in 5% of each specific probiotic group, compared to 30% of the placebo group	0.018	Cremonini *et al*.^47^
*H. pylori* therapy-associated side effects	88	DBRCT	*L. acidophilus* LA-5, *B. animalis* subsp. *lactis* Bb12, *Streptococcus salivarius* subsp. *thermophilus* (ABT-21 culture)Fermented milk, 125 g b.i.d. (≥1 × 10^6^ CFU/g of each strain) for 5 weeks (eradication triple therapy during fifth week of study intervention)	*H. pylori* eradication therapy-associated diarrhoea episodes (≥3 watery stools per day, with ≥1 day in the eradication week)	Number of diarrhoea episodes: active specific probiotic, 4; pasteurised specific probiotic, 2; acidified milk, 3Number of days with watery stools: active specific probiotic, 4; pasteurised specific probiotic, 10; acidified milk, 10Mean duration of diarrhoea episodes: active specific probiotic, 1.0 day; pasteurised specific probiotic, 5.0 days; acidified milk, 4.7 days	>0.05<0.05<0.05	de Vrese *et al*.^48^
*H. pylori* therapy-associated side effects	106	DBRCT	*Bacillus clausii* strains O/C, N/R, T and SIN (Enterogermina)One vial (each vial contains 2 × 10^9^ spores of *Bacillus clausii*) t.i.d., taken during 1 week of *H. pylori* eradication therapy and continued for 1 further week	*H. pylori* eradication therapy-associated side effects	Incidence of diarrhoea after 1 week: specific probiotic, 9.3%; placebo, 30.8%; RR: 0.30. Mean intensity and frequency of diarrhoea episodes were also reduced	<0.01	Nista *et al*.^49^
Functional GI symptoms	87	TBRCT	*B. animalis* subsp. *lactis* HN019 (HOWARU Bifido/DR10)Capsules, two dose groups: 1.8 billion or 17.2 billion CFU/day for 2 weeks	Whole-gut transit time	Change from baseline: specific probiotic groups, −25% and −33%; placebo group, +17%	<0.001	Waller *et al*.^50^
Women with mild digestive symptoms	197	DBRCT	*B. animalis* subsp. *lactis* DN-173 010 (Activia)Fermented milk, 125 g (1.25 × 10^10^ CFU) b.i.d. for 4 weeks	GI well-being	Proportion reporting improved GI well-being at weeks 1–4: specific probiotic, 37–41%; placebo, 22–34%; OR, 1.7	0.006	Guyonnet *et al*.^51^
Healthy, postprandial intestinal gas-related symptoms	61	DBRCT	*Bacillus coagulans* GBI-30, 6086 (Digestive Advantage Gas Defense Formula)One capsule (2 × 10^9^ CFU) o.i.d. for 4 weeks	GSRS abdominal pain, distension and flatus subscores, and SODA bloating and gas subscores	Difference between specific probiotic and placebo groups at 4 weeks:Abdominal pain (GSRS), −0.627Abdominal distension (GSRS), −0.572Flatus (GSRS), −0.511Bloating (SODA): −0.229Gas (SODA): − 0.348	0.0460.0610.1540.2940.118	Kalman *et al*.^52^
Lactose-intolerant individuals	60	RCT	*L. reuteri* DSM17938 (Reuterin)Two pills b.i.d. (4 × 10^8^ CFU/day) for 10 days preceding lactose breath test	Lactose breath test normalisation rate	N/A – primary end point data were not GI symptoms/HRQoL measures (bloating, abdominal pain, flatus and diarrhoea were assessed as secondary end points)	–	Ojetti *et al*.^53^
Elderly nursing home residents	179	DBRCT	(1) *B. longum* subsp*. longum* 46 and 2C, (2) *B. animalis* subsp. *lactis* Bb12 (Yosa)Fermented oat drinks, 1 × 10^9^ CFU/day (200 mL) for 7 months	Proportion of participants with bowel functioning on >30% of days	Bowel functioning on ≥30% of days: placebo, 49%specific probiotic 1, 70%specific probiotic 2, 59%Normal bowel functioning (solid or normal consistency of stools) on ≥30 days: placebo, 14%specific probiotic 1, 37%specific probiotic 2, 30%	0.0440.2530.0200.036	Pitkala *et al*.^54^
Healthy individuals (competitive cyclists)	88	DBRCT	*L. fermentum* VRI-003 (PCC) (ProBioPCC)One capsule (≥1 × 10^9^ CFU) o.i.d. for 11 weeks (mean)	Self-reported upper respiratory tract and GI symptoms	GI symptoms Ratio (99% CI) of number of episodes (specific probiotic/placebo): men, 2.06 (0.51–11); women, 3.02 (0.76–17)Ratio (99% CI) of duration of episodes (specific probiotic/placebo): men, 2.57 (0.53–17); women, 1.85 (0.35–27)Difference (99% CI) in severity (specific probiotic − placebo): men, −0.47 (−1.21 to 0.28); women, −0.31 (−1.39 to 0.79)Upper respiratory tract symptoms: no clear difference between groups	–	West *et al*.^55^
**Investigative strains**							
IBS (mild-to-moderate; Rome III)	122	DBRCT	*B. bifidum* MIMBb75One capsule (1 × 10^9^ CFU) o.i.d. for 4 weeks	Global IBS symptoms (7-point Likert scale)	Improvement from baseline: specific probiotic, −0.88; placebo, −0.16	<0.0001	Guglielmetti *et al*.^56^
IBS (Rome III)	70	DBRCT	*B. bifidum* BGN4, *B. animalis* subsp. *lactis* AD011, *L. acidophilus* AD031, *L. paracasei* subsp*. paracasei* IBS041One sachet b.i.d. (10 billion bacteria of each strain/day) for 8 weeks	Abdominal pain, flatus, defecation discomfort individual and sum scores	Abdominal pain (vs. baseline): Week 4: specific probiotic, −23.9; placebo, −10.9Week 8: specific probiotic, −31.9; placebo, −17.7Flatus (vs. baseline): Week 4: specific probiotic, −18.5; placebo, −18.4Week 8: specific probiotic, −27.0; placebo, −21.3 Defecation discomfort (vs. baseline): Week 4: specific probiotic, −29.2; placebo, −13.5Week 8: specific probiotic, −30.5; placebo, −18.4Sum score (vs. baseline): Week 4: specific probiotic, −71.7; placebo, −42.8Week 8: specific probiotic, −89.5; placebo, −57.5	0.0610.0450.9820.4370.0430.1220.1150.064	Hong *et al*.^57^
IBS (Rome I)	81	DBRCT	*L. rhamnosus* GG*, L. rhamnosus* Lc705, *Propionibacterium freudenreichii* subsp. *shermanii* JS, *B. breve* Bb99One capsule (8–9 × 10^9^ CFU total; equal amount of each strain) o.i.d. for 6 months	Change in weekly sum score of each of four symptoms (abdominal pain, distension, flatus and borborygmi), total symptom score and bowel habit	Baseline-adjusted symptom score, difference between specific probiotic and placebo: Abdominal pain: −1.5Distension: −1.6 Flatus: −1.2 Borborygmi: −2.2Total symptom score: −6.5Weekly defecation frequency: 1.3	0.1100.0830.2320.0080.0370.102	Kajander *et al*.^58^
IBS (Rome II)	16	DBRCT, crossover	*L. plantarum* MF1298One capsule (1 × 10^10^ CFU) o.i.d. for 3 weeks	Treatment preference	13 participants (81%) preferred placebo to the specific probiotic	0.012	Ligaarden *et al*.^59^
IBS (Rome III)	40	SBRCT	*L. acidophilus*-SDC 2012, *L. acidophilus*-SDC 2013 2 × 10^9^ CFU/mL in one capsule taken b.i.d. for 4 weeks	Abdominal pain (responder rate)	Proportion with improvement in abdominal pain/discomfort: specific probiotic, 80%; placebo, 35%	0.011	Sinn *et al*.^60^
Functional GI disorders	72	DBRCT	(1) *L. acidophilus*,*B. bifidum*,*Bacillus subtilis*,*L. delbrueckii* subsp. *bulgaricus*,*L. delbrueckii* subsp. *lactis* and *Bacillus lichenformis*, or (2) *L. acidophilus*,*B. bifidum*,*L. delbrueckii* subsp. *bulgaricus*,*L. delbrueckii* subsp. *lactis*,*L. brevis*,*L. caucasicus* (*nomina rejicienda*; now *L. delbrueckii* subsp. *delbrueckii*), *L. fermentum*,*L. leichmanii*,*L. paracasei* subsp. *paracasei*,*L. plantarum*,*L. helveticus* and *Saccharomyces boulardii*Caplets, each containing 5 × 10^7^ bacteria, taken for12 weeks (Week 1: one caplet od; Week 2: one caplet t.i.d.; Week 3: two caplets t.i.d.; Week 4: three caplets t.i.d.; Weeks 5–12: four caplets t.i.d.)	HRQoL (GIQLI)	GIQLI total score and well-being subscales (physical, social and mental) showed no significant change from baseline at 4, 8 and 12 weeks	>0.05	Kim *et al*.^61^
Healthy young adults	71	DBRCT	*B. animalis* subsp. *lactis* Bb12, *L. paracasei* subsp. *paracasei* CRL-431Capsules, four dose groups: 1 × 10^8^, 10^9^, 10^10^ or 10^11^ CFU o.i.d. for 3 weeks	Granulocyte activity	N/A – primary end point data were not GI symptoms/HRQoL measures (bowel habit was assessed as a secondary end point)	–	Larsen *et al*.^62^

AAD, antibiotic-associated diarrhoea; b.i.d., twice daily; CFU, colony forming units; CI, confidence interval; DBRCT double-blind randomised controlled trial; FDDQOL, Functional Digestive Disorders Quality of Life; GI, gastrointestinal; GIQLI, Gastrointestinal Quality of Life Index; GSRS, Gastrointestinal Symptom Rating Scale; HRQoL, health-related quality of life; IBS, irritable bowel syndrome; IBS-C, constipation-predominant IBS; IBS-D, diarrhoea-predominant IBS; o.i.d., once daily; OR, odds ratio; N/A, not applicable; RCT, randomised controlled trial; RR, relative risk; SBRCT, single-blind randomised controlled trial; SODA, Severity of Dyspepsia Assessment; SSI, Symptom Severity Index; TBRCT, triple-blind randomised controlled trial; t.i.d., three times daily.

* In some cases, the specific strain was not identified in the publication and could not be found elsewhere (e.g. it may be proprietary information).

† This study tested a third product (Ferzym) that was excluded from the current analysis because online information indicated that it was a synbiotic.

**Figure 2:**
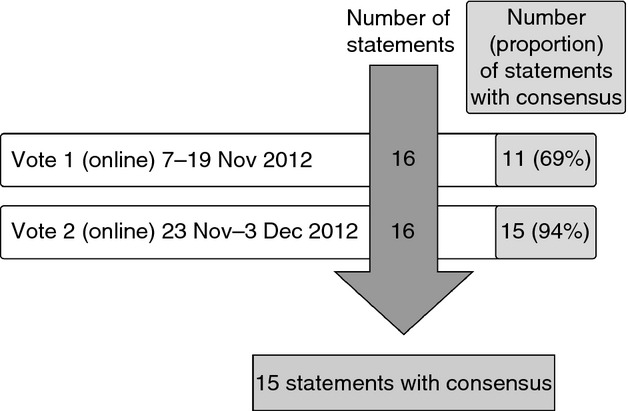
Overview of Delphi consensus development process and voting results.

Sixteen statements were developed, covering nine symptoms or problems plus general considerations relating to probiotic use. Of the 16 statements, 11 achieved consensus in the first round of voting and 15 achieved consensus in the second round (see Figure 2). Table 3 summarises the studies and specific probiotics with supportive or nonsupportive evidence for each consensus statement, together with an indication of whether the result was a primary or secondary end point, or part of a subanalysis. Table S1 shows probiotic availability in Europe, the USA and China.

**Table 3:** Overview of statements, grading and probiotics (marketed products and investigative strains) with supportive evidence. For many statements, the majority of the evidence came from populations with IBS, and the statements were therefore focused on IBS. Probiotics studied in indications other than IBS are still included below, but are placed in square brackets

Statement	Grade of evidence for effect	Level of agreement (%)	Probiotics for which studies show supportive evidence of benefit* – (bold font indicates primary end point data)	Probiotics for which studies suggest a lack of significant benefit* –(bold font indicates primary end point data)
**1: Specific probiotics help relieve overall symptom burden in some patients with IBS**	High	100	***Bifidobacterium bifidum*** **MIMBb75,**^56^ *B. longum* subsp. *infantis* 35624 (Bifantis/Align),^33^ ***Escherichia coli*** **DSM17252 (Symbioflor-2),**^27^ investigative combinations (BIFIDO,^57^ **Valio Bb99**^58^), marketed combinations **(Gefilus MAX,**^28^ **LAB4**^34^)	*Lactobacillus plantarum* MF1298,^59^ marketed combinations (**Cultura,**^31^–^32^ **Lactibiane**^26^)
**2: Specific probiotics may help relieve overall symptom burden in some patients with IBS-C**	Low	80	*B. animalis* subsp. *lactis* DN-173 010 (Activia)^35^–^36^	*B. longum* subsp. *infantis* 35624 (Bifantis/Align)^33^
**3: Specific probiotics help relieve overall symptom burden in some patients with IBS-D**	Moderate	100	*B. longum* subsp. *infantis* 35624 (Bifantis/Align),^33^ marketed combinations (AB100 Jianneng,^39^ **Duolac7**^37^)	Marketed combination **(VSL#3)**^38^
**4: Specific probiotics help reduce abdominal pain in some patients with IBS**	High	100	**[*****Bacillus coagulans*** **GBI-30, 6086 (Digestive Advantage Gas Defense Formula)**^52^**],** *B. animalis* subsp. *lactis* DN-173 010 (Activia),^35^ [*B. animalis* subsp. *lactis* HN019 (HOWARU Bifido/DR10)^50^], *B. bifidum* MIMBb75,^56^ ***B**. **longum*** **subsp.** ***infantis*** **35624 (Bifantis/Align),**^33^ ***Escherichia coli*** **DSM17252 (Symbioflor-2),**^27^ investigative combinations (**BIFIDO,**^57^ **SDC,**^60^ Valio Bb99^58^), [*L. reuteri* DSM17938 (Reuterin)^53^], marketed combinations (AB100 Jianneng,^39^ Lactibiane^26^)	*B. animalis* subsp. *lactis* DN-173 010 (Activia),^36^ [*B. animalis* subsp. *lactis* DN-173 010 (Activia)^51^], investigative combinations ([GoL6^61^], [GoL12^61^]), *L. plantarum* MF1298,^59^ marketed combinations (Cultura,^31^–^32^ Duolac7,^37^ Gefilus MAX,^28^ LAB4,^34^ VSL#3^29^–^38^)
**5: Specific probiotics help reduce bloating/distension in some patients with IBS**	Moderate	70	***B**. **animalis*** **subsp.** ***lactis*** **DN-173 010 (Activia),**^35^ *B. animalis* subsp. *lactis* DN-173 010 (Activia),^36^ *B. bifidum* MIMBb75,^56^ *B. longum* subsp. *infantis* 35624 (Bifantis/Align),^33^ *Escherichia coli* DSM17252 (Symbioflor-2),^27^ [*L. reuteri* DSM17938 (Reuterin)^53^], marketed combinations (Gefilus MAX,^28^ LAB4^34^)	**[*****Bacillus coagulans*** **GBI-30, 6086 (Digestive Advantage Gas Defense Formula)**^52^**],** [*B. animalis* subsp. *lactis* DN-173 010 (Activia)^51^], investigative combinations ([GoL6^61^], [GoL12^61^], **Valio Bb99**^58^), *L. plantarum* MF1298,^59^ marketed combinations (AB100 Jianneng,^39^ Cultura,^31^–^32^ Duolac7,^37^ **VSL#3,**^29^ VSL#3^38^)
**6: Probiotics tested to date do not reduce flatus in patients with IBS**	Low	90	[*B. animalis* subsp. *lactis* DN-173 010 (Activia)^51^], [*B. animalis* subsp. *lactis* HN019 (HOWARU Bifido/DR10)^50^], *B. longum* subsp. *infantis* 35624 (Bifantis/Align),^33^ [*L. reuteri* DSM17938 (Reuterin)^53^], marketed combinations (AB100 Jianneng,^39^ VSL#3^29^)	**[*****Bacillus coagulans*** **GBI-30, 6086 (Digestive Advantage Gas Defense Formula)**^52^**],** *B. animalis* subsp. *lactis* DN-173 010 (Activia),^35^ investigative combinations (**BIFIDO,**^57^ [GoL6^61^], [GoL12^61^], **Valio Bb99**^58^), ***L**. **plantarum*** **299v (ProViva),**^30^ marketed combinations (Duolac7,^37^ Gefilus MAX,^28^ VSL#3^38^)
**7: Specific probiotics may help reduce constipation in some patients with IBS**	Low	60 (no consensus)	*B. animalis* subsp. *lactis* DN-173 010 (Activia),^35^ [*B. animalis* subsp. *lactis* HN019 (HOWARU Bifido/DR10)^50^]	*B. bifidum* MIMBb75,^56^ investigative combinations ([GoL6^61^], [GoL12^61^])
**8: Specific probiotics help improve frequency and/or consistency of bowel movements in some patients with IBS**	Moderate	70	**[*****B**. **animalis*** **subsp.** ***lactis*** **Bb12 (Yosa)**^54^**],** *B. animalis* subsp. *lactis* DN-173 010 (Activia),^35^–^36^ [*B. animalis* subsp. *lactis* DN-173 010 (Activia)^51^], **[*****B**. **animalis*** **subsp.** ***lactis*** **HN019 (HOWARU Bifido/DR10)**^50^**],** *B. bifidum* MIMBb75,^56^ *B. longum* subsp. *infantis* 35624 (Bifantis/Align),^33^ *Escherichia coli* DSM17252 (Symbioflor-2),^27^ investigative combinations ([**Bioferme**^54^], [CH^62^], SDC,^60^ Valio Bb99^58^), marketed combinations (Duolac7,^37^ LAB4,^34^ Lactibiane^26^)	Investigative combination (BIFIDO^57^), *L. plantarum* MF1298^59^, marketed combinations (Cultura,^31^–^32^ Gefilus MAX,^28^ **VSL#3,**^38^ VSL#3^29^)
**9: Probiotics tested to date do not reduce diarrhoea in patients with IBS**	Very low	80	[*L. reuteri* DSM17938 (Reuterin)^53^]	[*B. animalis* subsp. *lactis* Bb12 (Yosa)^54^], [*B. animalis* subsp. *lactis* HN019 (HOWARU Bifido/DR10)^50^], investigative combinations ([Bioferme^54^], [GoL6^61^], [GoL12^61^]), *L. plantarum* MF1298,^59^ marketed combinations (Duolac7,^37^ Gefilus MAX^28^)
**10: In patients receiving antibiotic therapy, specific probiotics are helpful as adjuvant therapy to prevent, or reduce the duration of, associated diarrhoea**	High	100	***L**. **paracasei*** **subsp*****. paracasei*** **DN-114 001 (Actimel),**^43^ **marketed combination (Bio-K+ CL1285)**^40^,^41^	Marketed combinations (LAB4,^44^ **Lacidofil cap – underpowered**^45^)
**11: In patients receiving** ***Helicobacter pylori*** **eradication therapy, specific probiotics are helpful as adjuvant therapy to prevent or reduce the duration/intensity of associated diarrhoea**	High	100	***L**. **rhamnosus*** **GG (Giflorex),**^47^ **marketed combinations (ABT-21 culture,**^48^ **Enterogermina**^49^**),** ***Saccharomyces boulardii*** **(Codex,**^47^ **Reflor**^46^**)**	
**12: With specific probiotics, improvement of symptoms has been shown to lead to improvement in some aspects of health-related quality of life**	Moderate	80	***B**. **animalis*** **subsp.** ***lactis*** **DN-173 010 (Activia),**^51^ *B. animalis* subsp. *lactis* DN-173 010 (Activia),^36^ *B. bifidum* MIMBb75,^56^ *Escherichia coli* DSM17252 (Symbioflor-2),^27^ investigative combination (GoL12),^61^ marketed combinations (Duolac7,^37^ LAB4,^34^ Lactibiane^26^)	*Bacillus coagulans* GBI-30, 6086 (Digestive Advantage Gas Defense Formula),^52^ *B. longum* subsp. *infantis* 35624 (Bifantis/Align),^33^ investigative combinations (BIFIDO,^57^ **GoL6,**^61^ Valio Bb99^58^), marketed combinations (Cultura,^31^–^32^ Gefilus MAX^28^)
**13: Probiotics have a favourable safety profile in patients with a range of lower GI symptoms typically managed in primary care or general practice**	High	100	*B. animalis* subsp. *lactis* DN-173 010 (Activia),^36^ *B. animalis* subsp. *lactis* HN019 (HOWARU Bifido/DR10),^50^ *B. bifidum* MIMBb75,^56^ *B. longum* subsp. *infantis* 35624 (Bifantis/Align),^33^ investigative combinations (BIFIDO,^57^ CH,^62^ GoL6,^61^ GoL12,^61^ SDC,^60^ Valio Bb99^58^), *L. paracasei* subsp*. paracasei* DN-114 001 (Actimel),^43^ *L. rhamnosus* GG (Giflorex),^47^ marketed combinations (AB100 Jianneng,^39^ ABT-21 culture,^48^ Bio-K+ CL1285,^40^,^41^ Cultura, Duolac7,^37^ Gefilus MAX,^28^ LAB4,^34^ Lacidofil cap,^45^ VSL#3^29^–^38^), *Saccharomyces boulardii* (Codex,^47^ Reflor^46^)	*Escherichia coli* DSM17252 (Symbioflor-2),^27^ *L. fermentum* VRI-003 PCC,^55^ *L. plantarum* MF1298^59^
**14: Specific probiotics have a role in the management of some IBS symptoms and can also be used as an adjunct to conventional treatment**	NA	90	**–**	**–**
**15: Probiotic strains should be selected based on the patient's symptoms, the clinical indication and the available evidence; no probiotic alleviates the full range of symptoms in IBS**	NA	80	–	**–**
**16: When trying a probiotic therapy for a chronic GI problem, the product should be taken for 1 month; dose selection should be based on available evidence and manufacturers' recommendations**	NA	80	**–**	**–**

IBS, irritable bowel syndrome; IBS-C, constipation-predominant IBS; IBS-D, diarrhoea-predominant IBS.

* For simplicity, single-strain probiotics are identified by the name of the strain (and the brand name where available), and multi-strain products are identified as ‘combination (X)’ and listed in full below.

**Investigative combinations:** Valio Bb99: *Lactobacillus rhamnosus* GG, *L. rhamnosus* Lc705, *Propionibacterium freudenreichii* subsp. *shermanii* JS and *Bifidobacterium breve* Bb99 (Valio Ltd, Helsinki, Finland).

BIFIDO: *Bifidobacterium bifidum* BGN4, *B. animalis* subsp. *lactis* AD011, *Lactobacillus acidophilus* AD031 and *L. paracasei* subsp. *paracasei* IBS041 (BIFIDO Co. Ltd, Hongchun, Korea).

Bioferme: *Bifidobacterium longum* subsp. *longum* 46 and *B. longum* subsp. *longum* 2C (Bioferme Ltd, Kaarina, Finland).

CH: *Bifidobacterium animalis* subsp. *lactis* Bb12 and *Lactobacillus paracasei* subsp. *paracasei* CRL-431 (Chr. Hansen A/S, Hoersholm, Denmark).

GoL6 contains nonspecified strains from the species: *Lactobacillus acidophilus*,*Bifidobacterium bifidum*,*Bacillus subtilis*,*L. delbrueckii* subsp. *bulgaricus*,*L. delbrueckii* subsp. *lactis* and *Bacillus lichenformis* (Garden of Life, West Palm, FL, USA).

GoL12 contains nonspecified strains from the species: *Lactobacillus acidophilus, Bifidobacterium bifidum, L. delbrueckii* subsp. *bulgaricus*,*L. delbrueckii* subsp. *lactis*,*L. brevis*,*L. caucasicus* (*nomina rejicienda*; now *L. delbrueckii* subsp. *delbrueckii*)*, L. fermentum, L. leichmanii, L. paracasei* subsp. *paracasei, L. plantarum, L. helveticus* and *Saccharomyces boulardii* (Garden of Life, West Palm, FL, USA).

SDC: *Lactobacillus acidophilus*-SDC 2012 and *L. acidophilus*-SDC 2013 (Seoul Dairy Cooperative, Seoul, Korea).

**Marketed combinations:** AB100 Jianneng: *Streptococcus salivarius* subsp. *thermophilus*,*Lactobacillus delbrueckii* subsp. *bulgaricus*,*L. acidophilus* and *Bifidobacterium longum* subsp*. longum* (Bright Dairy, Shanghai, China).

ABT-21 culture: *Lactobacillus acidophilus* LA-5, *Bifidobacterium animalis* subsp. *lactis* Bb12 and *Streptococcus salivarius* subsp. *thermophilus* (Christian Hansen, Nienburg, Germany).

Bio-K+ CL1285: *Lactobacillus acidophilus* CL1285 and *L. paracasei* subsp*. paracasei* LBC80R (Bio-K+ International Inc., Quebec, QC, Canada).

Cultura: *Lactobacillus paracasei* subsp. *paracasei* F19, *L. acidophilus* La5 and *Bifidobacterium animalis* subsp. *lactis* Bb12 (Arla Foods Innovation, Stockholm, Sweden).

Duolac7: *Lactobacillus acidophilus* LH5*, L. plantarum* LP1*, L. rhamnosus* LR3, *Bifidobacterium breve* BR2, *B. animalis* subsp. *lactis* BL2, *B. longum* subsp*. longum* BG3 and *Streptococcus salivarius* subsp. *thermophilus* ST3 (Cell Biotech, Co. Ltd, Seoul, Korea).

Enterogermina: *Bacillus clausii* strains O/C, N/R, T and SIN (Sanofi Synthelabo OTC, Milan, Italy).

Gefilus MAX: *Lactobacillus rhamnosus* GG, *L. rhamnosus* Lc705, *Propionibacterium freudenreichii* subsp. *shermanii* JS and *Bifidobacterium animalis* subsp. *lactis* Bb12 (Valio Ltd, Helsinki, Finland).

LAB4: *Lactobacillus acidophilus* (CUL60 and CUL21), *Bifidobacterium animalis* subsp. *lactis* CUL34 and *B. bifidum* CUL20 (Cultech, Port Talbot, UK).

Lacidofil cap: *Lactobacillus rhamnosus* R0011 and *L. acidophilus* R0052 (Lallemand Inc., Montreal, QC, Canada).

Lactibiane: *Bifidobacterium longum* subsp. *longum* LA 101, *Lactobacillus acidophilus* LA 102, *L. delbrueckii* subsp. *lactis* LA 103 and *Streptococcus salivarius* subsp. *thermophilus* LA 104 (PiLeJe, Paris, France).

VSL#3: *Bifidobacterium longum* subsp*. longum, B. infanti*s subsp*. infantis*,*B. breve*,*Lactobacillus acidophilus*,*L. paracasei* subsp*. paracasei*,*L. delbrueckii* subsp. *bulgaricus*,*L. plantarum* and *Streptococcus salivarius* subsp. *thermophilus* (VSL Pharmaceuticals Inc., Gaithersburg, MD, USA).

For each consensus statement, the result of the second (final) vote and the grade of supporting evidence are given, followed by a discussion of the evidence. In some cases, the consensus statement is indication-specific; however, studies in other indications that provide relevant data are also described for completeness. In the following discussion, ‘significant’ refers to a statistically significant result (*P *< 0.05). Sometimes, a particular probiotic yielded conflicting results for a symptom/problem when it was investigated in different studies (see Table 3).

### Irritable bowel syndrome (global symptom assessment)

#### Statement 1: specific probiotics help relieve overall symptom burden in some patients with IBS

*Agreement*: 100% (6, 40%; 5, 50%; 4, 10%; grade of evidence for effect: *high*).

*Supportive evidence*: Eleven studies of 10 different probiotics evaluated overall symptoms in 1313 patients with IBS. Of these studies, nine evaluated overall IBS symptoms as a primary end point, with five reporting a significant beneficial effect of five different probiotic treatments compared with placebo^27^–^58^ and three reporting no significant differences between two specific probiotic treatments and placebo.^26^,^31^ One of the nine studies reported a significant improvement vs. placebo in a subanalysis of patients with a Bristol stool scale score of 3 or more at baseline, but no significant effect was seen in the overall study population.^57^ Two studies of two different probiotics evaluated overall IBS symptoms as a secondary end point only, with one reporting a negative effect of the specific probiotic treatment compared with placebo,^59^ and one dose-ranging study^33^ reporting a beneficial effect of the specific probiotic treatment at the 1 × 10^8^ CFU dose, but not at the lower and higher doses tested (1 × 10^6^ and 1 × 10^10 ^CFU).

#### Statement 2: specific probiotics may help relieve overall symptom burden in some patients with IBS-C

*Agreement*: 80% (6, 10%; 5, 30%; 4, 40%; 3, 10%; 2, 10%; grade of evidence for effect: *low*).

*Supportive evidence*: Three studies of two different probiotics evaluated overall IBS symptoms as a secondary end point in 376 patients with IBS-C. One study reported a beneficial effect of the specific probiotic treatment vs. placebo,^35^ and another study of the same probiotic reported a significant improvement from baseline in the probiotic group, but not in the placebo group, in a subanalysis of patients with fewer than three bowel movements/week.^36^ One study of a different probiotic reported no significant improvement in symptoms vs. placebo.^33^

#### Statement 3: specific probiotics help relieve overall symptom burden in some patients with IBS-D

*Agreement*: 100% (6, 10%; 5, 70%; 4, 20%; grade of evidence for effect: *moderate*).

*Supportive evidence*: Four studies of four different probiotics evaluated overall IBS symptoms in 305 patients with IBS-D. Two studies evaluated overall IBS symptoms as a primary end point, with one reporting a significant beneficial effect of the specific probiotic treatment compared with placebo,^37^ and one reporting no significant difference.^38^ Two studies evaluated overall IBS symptoms as a secondary end point only, and both reported a significant beneficial effect of the specific probiotic treatments^33^–^39^; one of these was a *post hoc* analysis of the most effective dose in a subset of patients with IBS-D.^33^

### Abdominal pain

#### Statement 4: specific probiotics help reduce abdominal pain in some patients with IBS

*Agreement*: 100% (6, 30%; 5, 50%; 4, 20%, grade of evidence for effect: *high*).

*Supportive evidence*: Eighteen studies of 15 different probiotics evaluated abdominal pain in 1806 patients with IBS. Of these studies, six (each examining a different probiotic) evaluated abdominal pain as a primary end point, with four showing a significant beneficial effect of specific probiotic treatments compared with placebo,^27^–^60^ one^58^ showing a trend towards a beneficial effect in the weekly symptom score for abdominal pain (in a secondary analysis, abdominal pain was reduced in a significantly greater proportion of the probiotic group than the placebo group) and one^26^ showing no significant increase in the proportion of patients reporting symptom relief, but a significantly greater decrease in the abdominal pain score in the probiotic group than the placebo group. Twelve studies evaluated abdominal pain as a secondary end point only. Results from these studies were mixed: one reported a negative effect of the specific probiotic treatment,^59^ eight (examining six different probiotics) reported no significant effect^28^–^38^ and three reported a significant beneficial effect of three different probiotics^35^,^39^ (one of which^35^ also showed no significant effect in another study^36^).

Abdominal pain was examined in indications other than IBS in five studies of six different probiotics. One study examined abdominal pain as a primary end point in individuals with symptoms related to postprandial intestinal gas, and found a significant improvement in the probiotic group compared with the placebo group.^52^ Four studies examined abdominal pain as a secondary end point only, with two reporting no significant difference between three different probiotic treatments and placebo,^51^–^61^ and two (examining one probiotic in lactose-intolerant individuals undergoing a hydrogen breath test^53^ and a different probiotic in patients with functional GI symptoms^50^) reporting significantly improved abdominal pain vs. baseline in the probiotic group, but not in the placebo group.

### Bloating/distension

#### Statement 5: specific probiotics help reduce bloating/distension in some patients with IBS

*Agreement*: 70% (6, 10%; 5, 30%; 4, 30%; 3, 20%; 2, 10%; grade of evidence for effect: *moderate*).

*Supportive evidence*: Fifteen studies of 12 different probiotics evaluated treatment of bloating/distension in 1596 patients with IBS. Of these studies, three (examining three different probiotics) evaluated bloating/distension as a primary end point, with one reporting a significant, beneficial effect of the specific probiotic treatment vs. placebo,^35^ and two reporting no significant differences.^29^–^58^ Twelve studies evaluated bloating/distension as a secondary end point only, with six reporting a significant, beneficial effect of six different probiotic treatments^27^–^56^ (one of which^36^ also showed a beneficial effect as a primary end point in another study^35^). In one study,^36^ the significant effect was seen at week 3, but not at week 6, and in another,^33^ the significant effect was seen at one specific dose only. The remaining six studies reported no significant difference between five different probiotic treatments and placebo^31^–^59^ (one of these probiotics^38^ also showed no significant effect as a primary end point^29^).

Four studies investigated the effect of five different probiotics on distension/bloating in indications other than IBS. One study evaluated symptoms related to postprandial intestinal gas as a primary end point in healthy individuals and reported no significant differences between the probiotic and placebo groups.^52^ The remaining three studies (of four different probiotics) evaluated distension/bloating as a secondary end point, with single studies reporting no significant differences between the probiotic and control groups in women with mild digestive symptoms^51^ and patients with FGID.^61^ The third study, in individuals with lactose intolerance undergoing a hydrogen breath test, reported significantly reduced bloating in the group receiving the specific probiotic treatment, but no significant improvement in the placebo group.^53^

### Flatus

#### Statement 6: probiotics tested to date do not help reduce flatus in patients with IBS

*Agreement*: 90% (6, 20%; 5, 30%; 4, 40%; 2, 10%; grade of evidence for effect: *low*).

*Supportive evidence*: Overall, 10 studies, using nine different probiotics, evaluated flatus in 797 patients with IBS. The statement on flatus had to be formulated in the negative as there was a low level of agreement when it was formed in the positive. This was because the evidence in IBS studies was weak: all three studies that examined flatus as a primary end point,^30^,^57^ and four of seven studies in which flatus was a secondary end point only,^28^–^38^ showed no significant difference between seven specific probiotic treatments and control. The remaining three studies that evaluated flatus as a secondary end point reported a significant beneficial effect of three different probiotic treatments^29^,^33^ (one of which^29^ also showed no significant effect in another study^38^). In one of these studies,^33^ the significant effect was seen at one specific dose only.

Five studies examined the effect of six different probiotics on flatus in indications other than IBS. In two studies, no significant effects on flatus (primary end point for one probiotic^52^; secondary end point for two other probiotics^61^) were reported. In three studies, a significant benefit of three different probiotic treatments on flatus (secondary end point) was noted; these studies were in women with mild digestive symptoms,^51^ patients with functional GI symptoms^50^ and individuals with lactose intolerance undergoing a lactose breath test.^53^

### Constipation

#### Statement 7: specific probiotics may help reduce constipation in some patients with IBS

*Agreement*: 60% (5, 20%; 4, 40%; 3, 30%; 1, 10%; grade of evidence for effect: *low*).

*Supportive evidence*: Two studies of two different probiotics examined treatment of constipation as a secondary end point in 156 patients with IBS. One study (specifically in patients with IBS-C) reported significant improvements with the specific probiotic treatment vs. control for some of the end points (orocaecal transit time, colonic transit time and urgency), but not others (stool frequency and consistency, straining during evacuation and feelings of incomplete evacuation).^35^ The second study did not detect any effects of the specific probiotic treatment on the frequency of bowel movements and feelings of incomplete evacuation.^56^

Two studies of three different probiotics examined constipation in patients with broader FGID. Of these studies, one^61^ reported no significant effect of two different probiotic treatments, and the other study^50^ did not report a between-group statistical analysis; however, the decrease in constipation frequency score was approximately twofold greater in the probiotic groups than in the placebo groups.

### Bowel habit

#### Statement 8: specific probiotics help improve frequency and/or consistency of bowel movements in some patients with IBS

*Agreement*: 70% (6, 10%; 5, 40%; 4, 20%; 3, 20%; 2, 10%; grade of evidence for effect: *moderate*).

*Supportive evidence*: Seventeen studies of 14 different probiotics evaluated bowel habit in 1777 patients with IBS. Of these, two studies of two different probiotics evaluated bowel habit as a primary end point, with one study reporting no difference in GI transit measurements between the probiotic and placebo groups,^38^ and one reporting no significant difference in weekly defecation frequency between the probiotic and placebo groups, but a significant positive effect of the specific probiotic treatment vs. placebo on the secondary end points of urgency and feelings of incomplete evacuation.^58^

Fifteen of the 17 studies in patients with IBS evaluated bowel habit as a secondary end point only. The main end points assessed were stool frequency, stool consistency and satisfaction with bowel habits. One or more of these end points were evaluated in 14 studies, with seven reporting significant beneficial effects of seven different probiotics,^26^–^56^ six reporting no significant effects of five different probiotics^28^–^60^ (one of which^29^ showed no significant benefit as a primary end point in another study^38^) and one reporting a significant negative effect of the specific probiotic treatment.^59^ In addition, one study reported significant improvements in the secondary end points of transit time (see section) and urgency in patients with IBS-C, but no significant effects on straining and feelings of incomplete evacuation.^35^

Four studies of five different probiotic treatments assessed bowel habit in indications other than IBS, with all five probiotics showing significant effects on measures of bowel habit (see Table 3).^50^–^62^

### Diarrhoea

#### Statement 9: probiotics tested to date do not reduce diarrhoea in patients with IBS

*Agreement*: 80% (6, 30%; 5, 30%; 4, 20%; 2, 20%; grade of evidence for effect: *very low*).

*Supportive evidence*: Three studies of three different probiotics evaluated, as a secondary end point, the treatment of diarrhoea in 152 patients with IBS. Two studies reported no difference between specific probiotic treatments and placebo,^28^–^37^ and one study reported a significant worsening of diarrhoea with the specific probiotic treatment compared with placebo.^59^

Four studies of six different probiotics evaluated diarrhoea as a secondary end point in indications other than IBS. Specific probiotic treatment had no significant effect on diarrhoea in elderly nursing home residents,^54^ individuals with a functional bowel disorder^61^ and individuals with functional GI symptoms.^50^ The only identified study to show a beneficial effect was a study of one specific probiotic in patients with lactose intolerance^53^; in this study, diarrhoea improved significantly in the probiotic group, but not in the placebo group.

#### Statement 10: in patients receiving antibiotic therapy, specific probiotics are helpful as adjuvant therapy to prevent or reduce the duration of associated diarrhoea

*Agreement*: 100% (6, 60%; 5, 40%; grade of evidence for effect: *high*).

*Supportive evidence*: Six studies of four different probiotics examined prevention of AAD and/or reduction in AAD in 1246 patients who received antibiotics. Although initiated in a hospital setting, these studies were included because of the relevance of AAD to primary care. Five studies examined AAD as a primary end point, with four studies of two different probiotics showing a significant reduction in AAD,^40^–^43^ and one underpowered study of another probiotic showing a nonsignificant reduction.^45^ One study assessed AAD as a secondary end point only and found no difference between the probiotic and placebo groups.^44^

#### Statement 11: in patients receiving *H. pylori* eradication therapy, specific probiotics are helpful as adjuvant therapy to prevent or reduce the duration/intensity of associated diarrhoea

*Agreement*: 100% (6, 60%; 5, 40%; grade of evidence for effect: *high*).

*Supportive evidence*: Four studies, which evaluated five different probiotics, had a primary objective to investigate the occurrence of diarrhoea as a side effect of *H. pylori* eradication triple therapy in 382 patients. All four studies reported a significant benefit of specific probiotic treatments compared with placebo.^46^–^49^ However, the results for two of the probiotic treatments were mixed, with a significant benefit of the specific probiotic treatment seen after 1 week, but not 2 weeks, in one study,^49^ and significantly fewer days with diarrhoea and shorter mean duration of diarrhoea episodes, but no significant difference in frequency of diarrhoea episodes, in the probiotic group compared with the placebo group in another study.^48^

### Health-related quality of life

#### Statement 12: with specific probiotics, improvement of symptoms has been shown to lead to improvement in some aspects of health-related quality of life

*Agreement*: 80% (6, 10%; 5, 30%; 4, 40%; 3, 20%; grade of evidence for effect: *moderate*).

*Supportive evidence*: Health-related quality of life was assessed as a primary end point in three studies of three different probiotics. One study in patients with IBS-C^36^ reported no significant difference between the probiotic and placebo groups for the change from baseline in the discomfort dimension score of the Functional Digestive Disorders Quality of Life (FDDQL) questionnaire (primary end point); however, the probiotic group had a significantly greater proportion of responders for the discomfort dimension score than the placebo group at week 3. Another study of the same probiotic was performed in women with minor GI symptoms, and reported a significantly greater improvement in ‘GI well-being’ (primary end point) in the probiotic group than in the placebo group.^51^ The remaining study assessed two different probiotics in patients with FGID^61^ and reported no significant differences between the probiotic and control groups for the Gastrointestinal Quality of Life Index (GIQLI) total score and well-being subscales (physical, social and mental; primary end point); however, the 36-item Short-Form Health Survey (SF-36; secondary end point) showed significant changes in the probiotic groups for physical functioning and/or ‘role–physical’ domains, but no significant changes in the control groups.

Twelve studies assessed aspects of health-related quality of life as secondary end points only. Of these, seven (evaluating six different probiotics) found no difference between treatment groups in measures of health-related quality of life.^28^–^58^ The remaining five studies (all in patients with IBS) reported significant benefits of five different probiotic treatments for some aspects of health-related quality of life.^26^–^56^

### Adverse events

#### Statement 13: probiotics have a favourable safety profile in patients with a range of lower GI symptoms typically managed in primary care or general practice

*Agreement*: 100% (6, 80%; 5, 20%; grade of evidence for effect: *high*).

*Supportive evidence*: Safety data were reported in 28 studies, none of which revealed significant treatment-emergent adverse events that were attributed to probiotic use. Of the 28 studies, 25 reported no relevant differences in safety between 23 specific probiotic treatments and placebo.^28^–^62^ The remaining three studies (each examining a different probiotic) are summarised below.

In a study of patients with IBS, two patients in the probiotic group discontinued from the study because of adverse events (moderate nausea and severe exanthema). However, the most frequent adverse events (fatigue, pruritus and diarrhoea) occurred equally in the probiotic and placebo groups.^27^ In a study of patients with IBS, one participant had a short stay in hospital for cervicobrachialgia 2 weeks after the end of the specific probiotic treatment; however, there was no organic explanation and the patient continued in the trial.^59^ In a study of healthy athletes, there was a twofold increase in the number and duration of mild GI symptoms in the probiotic group compared with the placebo group, although severity tended to be lower.^55^

### General considerations

#### Statement 14: specific probiotics have a role in the management of some IBS symptoms and can also be used as an adjunct to conventional treatment

*Agreement*: 90% (6, 60%; 5, 20%; 4, 10%; 2, 10%; grade of evidence for effect: *NA*).

Statement 14 was derived from the evidence collated during this international consensus and from the clinical experience of the Consensus Group. It was presented for voting with the following explanations: for patients with IBS who are responding positively to conventional therapy, probiotics should be considered as an adjunct rather than a replacement for conventional treatment; for patients with IBS who are not responding to conventional therapy, replacement of the ineffective conventional treatment with a probiotic may be considered.

#### Statement 15: probiotic strains should be selected based on the patient's symptoms, the clinical indication and the available evidence; no probiotic alleviates the full range of symptoms in IBS

*Agreement*: 80% (6, 20%; 5, 50%, 4, 10%; 3, 10%; 2, 10%; grade of evidence for effect: *NA*).

Statement 15 was based on the observation that some studies in patients with IBS showed a beneficial effect of a given probiotic on some symptoms, but not on others. For example, one study reported that *Bifidobacterium bifidum* MIMBb75 was beneficial for improving global IBS symptoms, bloating and aspects of health-related quality of life (physical and mental health), but not for frequency of bowel movement and feeling of incomplete bowel evacuation.^56^ In another study, *Bifidobacterium longum* subsp. *infantis* 35624 (1 × 10^8 ^CFU once daily) significantly improved all IBS symptoms assessed, except urgency.^33^ Studies of multi-strain probiotics provide further examples.^28^,^57^

#### Statement 16: when trying a probiotic therapy for a chronic GI problem, the product should be taken for 1 month; dose selection should be based on available evidence and manufacturers' recommendations

*Agreement*: 80% (6, 30%; 5, 40%; 4, 10%; 2, 20%; grade of evidence for effect: *NA*).

Statement 16 was based on the observation that the treatment duration was at least 4 weeks in most studies (21/24) that examined probiotics for the treatment of chronic GI problems.

## Discussion

This is the first practical consensus on the role of probiotics in the management of the full range of lower GI symptoms in adults consulting clinicians in a pragmatic setting (particularly in primary care). The outcome of this consensus (summarised in Tables 3 and 4) is relevant to both primary care physicians and gastroenterologists, and is important because patients as well as the general public are becoming increasingly aware of probiotics as a result of considerable media interest and intensive advertising campaigns. Consequently, there is a need for physicians to be in a position to provide advice on whether probiotics might be helpful for patients with specific lower GI symptoms/problems, and, if so, which ones might be appropriate to recommend. What is evident is that there is no clear, simple guidance possible and that research linking specific probiotics with particular symptoms or problems is complex to interpret, partly because of the widespread types of studies and end points. However, our research confirms that there is positive evidence for the role of probiotics in lower GI problems.

**Table 4:** Practical implications of consensus statements for physicians

Grade of evidence for effect	Symptoms/indications	Meaning for physicians
High	Overall symptoms and abdominal pain in IBSPrevention or reduction of diarrhoea in patients receiving antibiotics, including *Helicobacter pylori* eradication therapy	Probiotics with supportive evidence for benefit should be tried
Moderate	Overall symptoms in IBS-DBowel movements and bloating/distension in IBS	Probiotics with supportive evidence for benefit could be tried
Low	Overall symptoms in IBS-C	Probiotics with supportive evidence for benefit could be considered
Very low	Flatus in IBS*Diarrhoea in IBS	Currently no evidence to support use of probiotics

Constipation in IBS is not addressed in this table because consensus was not achieved for this statement.

IBS, irritable bowel syndrome; IBS-C, constipation-predominant IBS; IBS-D, diarrhoea-predominant IBS.

* The grade of evidence was initially deemed to be low (rather than very low) for flatus in IBS, but the statement was revised to be negative in response to voter feedback during the Delphi process.

### Consensus findings in comparison with other research

A strong consensus was reached on the positive role of probiotics in the prevention of AAD or diarrhoea associated with *H. pylori* eradication therapy. Although the strain-/formulation-specific properties of different probiotics mean that meta-analyses of probiotics should be interpreted with caution, our findings are consistent with several previous meta-analyses,^63^–^67^ and the potential of probiotics to reduce side effects is also noted in the Maastricht IV/Florence consensus report on the management of *H. pylori* infection.^68^ Furthermore, our finding is consistent with the proposed role of probiotics in maintaining the gut microbiota,^69^ which are typically disturbed during oral antibiotic treatment. However, the current consensus does not support a role for probiotics in the treatment of diarrhoea in adults with IBS. The role of probiotics in the prevention of traveller's diarrhoea is also a topic of interest,^70^–^71^ but was not addressed in any of the studies eligible for the current analysis.

The Consensus Group concluded, with a high level of evidence, that specific probiotics help reduce overall symptom burden and abdominal pain in IBS (Statements 1 and 4: 100% agreement among voters), consistent with previous meta-analyses and reviews.^2^,^8^ There was a moderate level of evidence, with 70% agreement, for a role of specific probiotics in reducing bloating and improving the frequency and/or consistency of bowel movements in IBS (Statements 5 and 8). The level of evidence was also moderate for a role of probiotics in improving some aspects of health-related quality of life (Statement 12: 80% agreement); there is a need for more research on the effects of probiotics on health-related quality of life, because this has clear implications for the day-to-day functioning of the patient.

There was a low level of evidence for a role of specific probiotics in reducing constipation [Statement 7: 60% agreement (no consensus)] and for the statement that probiotics tested to date do not reduce flatus in patients with IBS (Statement 6: 90% agreement). An overview of meta-analyses in IBS showed a similar trend, with more supportive evidence available for overall symptom burden and abdominal pain than for flatus.^8^ Nevertheless, a meta-analysis of studies in adults with IBS did report a significant reduction in flatus with probiotic treatment.^72^

The lack of consensus on the role of probiotics in the management of constipation is consistent with the World Gastroenterology Organisation guideline on prebiotics and probiotics, which recommends certain prebiotics, but not probiotics, for the treatment of constipation.^10^ The grade of evidence and level of agreement were higher for bowel habit than for constipation; a possible explanation is that improvement in individual measures of function might be easier to achieve (or measure) than improvement in multiple facets of a GI problem. However, other confounding factors may play a role: besides a potential gender effect, bifidobacteria, attaining their highest counts in the colon, are more likely to have an effect on the colonic transit time than lactobacilli, which occur mainly in the small bowel.

Oral probiotics had a favourable safety profile in the included studies overall; the majority of the studies found no differences in safety between probiotic and placebo, and none of the studies identified significant adverse events attributed to probiotic use. The statement on the safety of probiotics achieved the highest degree of consensus. However, safety should not be generalised to untested situations, including other probiotics and different modes of administration, such as delivery by enteral tube.^73^ In addition, their use in certain patient groups, such as those who are immuno-compromised, needs to be considered on a case-by-case basis – at present, there are few data on the safety of probiotics in such patients. Immune compromise (including a debilitated state or malignancy) has been identified as a risk factor for rare cases of bacteraemia or fungaemia in patients taking certain probiotics (most commonly *Saccharomyces boulardii*).^74^,^75^

### Strengths and limitations

Suboptimal trial design has been highlighted as an important issue in studies of probiotics.^77^ To address this, we applied strict quality criteria in the selection of papers: only randomised, placebo-controlled clinical trials of probiotics that had suitable follow-up were included in the analysis. However, a limitation of the current consensus, similar to any systematic review, is the potential for publication bias – inconclusive or negative results are less likely to be published than positive results. Furthermore, studies designed to assess one end point can show positive effects on other end points by chance. We have therefore only included studies with a sample size calculation and we have distinguished between results that were assessed as primary and secondary end points to limit the influence of chance findings in secondary end points. Challenges identified by the Consensus Group during the voting process included the small number of high-quality studies, small study populations and diverse results, with some members noting that the evidence was insufficient to fully support Statements 5 and 8, and that there is currently insufficient evidence to personalise choices in probiotic treatment (in response to Statement 15). The Consensus Group included representatives from many European countries, but the relevance of the statements to all European primary care/gastroenterology settings cannot be determined.

Factors determining the response to treatment include probiotic strain(s), dose and mode of administration, health status of the patient, diet and concomitant medications (e.g. antibiotics and antacids). The variable results noted across some of the studies included in our analysis could have been affected by any of these factors, as well as the different patient groups enrolled in the studies and different levels of placebo response. Furthermore, this consensus focused on adults, and the statements cannot be extended to children. Additional points to consider are that probiotic research is evolving rapidly, and that the current statements reflect physicians', rather than patients', perspectives. Many patients have an interest in probiotics and their potential to reduce their symptoms,^78^–^79^ and may take probiotics (or products incorrectly identified as probiotics) before consulting their physician. Therefore, educational materials for the general public are also needed to improve understanding and to ensure appropriate use of probiotics (see the consumer guidelines of the International Scientific Association for Probiotics and Prebiotics for an example^15^–^80^). To address these points, the ESPCG intends to update this consensus publication with new research and input from patient groups in 3 years.

Most of the studies eligible for inclusion in this consensus focused on IBS or AAD; only a small subset of the studies examined probiotics in healthy individuals or patients with lactose malabsorption, other functional GI problems or mild GI symptoms, and therefore specific statements were not prepared for these groups. Nevertheless, this small subset of studies was still included alongside the statements in Table 3 for completeness. The prophylactic application of probiotics is a potentially interesting area for future research, although it will be challenging in terms of study design.

## Conclusions and clinical implications

The practical clinical implications of the consensus statements are summarised for each grade of evidence in Table 4. It should be noted that effects are strain-/formulation-specific and cannot be extrapolated from one probiotic to another. Furthermore, specific probiotics will have different effects in different patients; a probiotic that does not work in one indication may have evidence supporting a beneficial effect in a different indication or for a different symptom. When trying a probiotic therapy for a chronic GI problem, it is critically important that the product is taken in adequate doses on a regular basis (e.g. just before a meal) for a reasonable period of time, which should be at least a month, unless it cannot be tolerated for any reason. Regular consumption is important because probiotic strains are transient and will generally be washed out within days, although strain-specific differences occur, for example, linked to the production of pili^81^–^82^ or mucus-binding proteins^83^ by the probiotic bacteria.

The need for objective, evidence-based guidance on the role of probiotics is becoming increasingly important as public awareness of probiotics grows. This consensus is intended as a practical reference to help physicians make appropriate, evidence-based recommendations to patients who might benefit from probiotic treatment. Overall, the randomised, placebo-controlled trials included in our analysis support, with a high evidence level, a role for specific probiotics in the management of overall symptoms and abdominal pain in patients with IBS, and for preventing or reducing diarrhoea in patients receiving antibiotics or *H. pylori* eradication triple therapy. The trials support, with a moderate evidence level, a role for specific probiotics in managing overall symptoms in patients with IBS-D; improving bowel movements and bloating/distension in patients with IBS; and improving some aspects of health-related quality of life.

## Authorship

*Guarantor of the article*: APSH initiated the project.

*Author contributions*: APSH, BP, CM, PW, CW, NdW, Lamya Moulay and Jane Mason developed the initial literature search strategy. APSH, BP, CM, PW, LA, PF, CL, JM, J-MPdF, GR, CW, NdW, Lamya Moulay, Jane Mason and Bohumil Seifert took part in the screening and analysis of the retrieved references. APSH, BP, CM, PW, LA, PF, CL, JM, J-MPdF, GR, CW, NdW and Bohumil Seifert attended a workshop at which the overall goals and process were agreed upon. APSH, BP, CM, PW, CW and NdW drafted statements. BP, PW, LA, PF, CL, JM, J-MPdF, GR, NdW and Bohumil Seifert voted on the statements. Paul Sinclair provided support and guidance on the Delphi voting process. CM, CW and Jane Mason prepared an outline and revised subsequent drafts of the paper. APSH, BP, PW, LA, PF, CL, JM, J-MPdF, GR and NdW revised the outline and subsequent drafts of the paper. All authors have approved the final version of the article, including the authorship list.

## SUPPORTING INFORMATION

Table S1 Probiotic availability by country in Europe, the USA and China.
